# Genome3D: integrating a collaborative data pipeline to expand the depth and breadth of consensus protein structure annotation

**DOI:** 10.1093/nar/gkz967

**Published:** 2019-11-16

**Authors:** Ian Sillitoe, Antonina Andreeva, Tom L Blundell, Daniel W A Buchan, Robert D Finn, Julian Gough, David Jones, Lawrence A Kelley, Typhaine Paysan-Lafosse, Su Datt Lam, Alexey G Murzin, Arun Prasad Pandurangan, Gustavo A Salazar, Marcin J Skwark, Michael J E Sternberg, Sameer Velankar, Christine Orengo

**Affiliations:** 1 Institute of Structural and Molecular Biology, UCL, Gower Street, London WC1E 6BT, UK; 2 MRC Laboratory of Molecular Biology, Francis Crick Avenue, Cambridge CB2 0QH, UK; 3 Department of Biochemistry, University of Cambridge, Old Addenbrooke’s Site, 80 Tennis Court Road, Cambridge CB2 0QH, UK; 4 Department of Computer Science, UCL, Gower Street, London WC1E 6BT, UK; 5 The Francis Crick Institute, 1 Midland Rd, London NW1 1AT, UK; 6 European Bioinformatics Institute, Wellcome Trust Genome Campus, Hinxton, Cambridgeshire CB10 1SD, UK; 7 Centre for Bioinformatics, Department of Life Sciences, Imperial College London, London, SW7 2AZ, UK; 8 Faculty of Science and Technology, Universiti Kebangsaan Malaysia, Bangi, Selangor 43600, Malaysia

## Abstract

Genome3D (https://www.genome3d.eu) is a freely available resource that provides consensus structural annotations for representative protein sequences taken from a selection of model organisms. Since the last NAR update in 2015, the method of data submission has been overhauled, with annotations now being ‘pushed’ to the database via an API. As a result, contributing groups are now able to manage their own structural annotations, making the resource more flexible and maintainable. The new submission protocol brings a number of additional benefits including: providing instant validation of data and avoiding the requirement to synchronise releases between resources. It also makes it possible to implement the submission of these structural annotations as an automated part of existing internal workflows. In turn, these improvements facilitate Genome3D being opened up to new prediction algorithms and groups. For the latest release of Genome3D (v2.1), the underlying dataset of sequences used as prediction targets has been updated using the latest reference proteomes available in UniProtKB. A number of new reference proteomes have also been added of particular interest to the wider scientific community: cow, pig, wheat and mycobacterium tuberculosis. These additions, along with improvements to the underlying predictions from contributing resources, has ensured that the number of annotations in Genome3D has nearly doubled since the last NAR update article. The new API has also been used to facilitate the dissemination of Genome3D data into InterPro, thereby widening the visibility of both the annotation data and annotation algorithms.

## INTRODUCTION

Detailed information about 3D structure can provide critical insight when attempting to understand the functional roles of proteins. Investigating mechanisms such as protein-protein binding, enzyme active sites and the functional implications of genetic mutations all benefit from information about the structural environment in which the mechanism occurs. A wealth of high resolution structural data is already available in the wwPDB (1), however this only represents a small amount (∼0.1%) of all the known protein sequences in resources such as UniProtKB (2) and Ensembl (3). In cases where experimental structural data is missing, structure prediction algorithms can be used to suggest structural features directly from protein sequence.

Genome3D is a collaborative resource that combines protein annotations from a number of structure prediction and structure classification groups (Blundell, Gough, Jones, Murzin, Orengo and Sternberg). Contributing groups identify homologous relationships between unannotated protein sequences in the shared dataset and structural domains from classification databases (SCOP (4) and CATH (5)). The Genome3D resource allows contributors to provide two types of submissions: structural annotations (predicting the location of a close relationship to a known structure) and 3D models (coordinates of the 3D model structure). All data is then collated, providing a consensus view of predictions for each protein sequence thus allowing similarities and differences between the predictions to be analysed (see Table 1).

**Table 1.** Summary of the resources currently contributing to Genome3D. Resources either contribute structural predictions (domain annotations and/or 3D models) or a domain classification scheme

Resource (reference)	Principal Investigator	Contribution	Classification source
DomSerf (8)	Jones	Prediction (3D Models)	CATH
FUGUE (9)	Blundell	Prediction (Domains)	CATH + SCOP
Gene3D (10)	Orengo	Prediction (3D Models + Domains)	CATH
pDomTHREADER (8)	Jones	Prediction (Domains)	CATH
PHYRE2 (11)	Sternberg / Kelley	Prediction (3D Models + Domains)	SCOP + PDB
SUPERFAMILY (12)	Gough	Prediction (3D Models + Domains)	SCOP
VIVACE	Blundell	Prediction (3D Models)	CATH + SCOP
CATH (5)	Orengo	Classification	-
SCOP (4)	Murzin	Classification	-

One of the key aims of Genome3D is to bring together structural annotations from a variety of different sources to make it possible for individual predictions to be appraised within a consensus context. For this consensus view to be useful, it should be immediately obvious to a user which features (e.g. domain boundaries, evolutionary relationships) are shared by all annotation methods and which features look more like outliers. Since annotations are based on relationships to CATH and SCOP structural domains, an important part of identifying similarities between the different annotation algorithms was identifying similarities between the underlying classification schemes. As such, Genome3D also provides carefully curated list of superfamilies identified as equivalent (or highly similar) between CATH and SCOP. For convenience, these equivalences have been organised into Bronze, Silver and Gold categories according to increasing similarity between the CATH and SCOP superfamily. The method is discussed in greater detail in previous Genome3D articles (6,7) and summarised in the improvements section.

**Figure 1.**
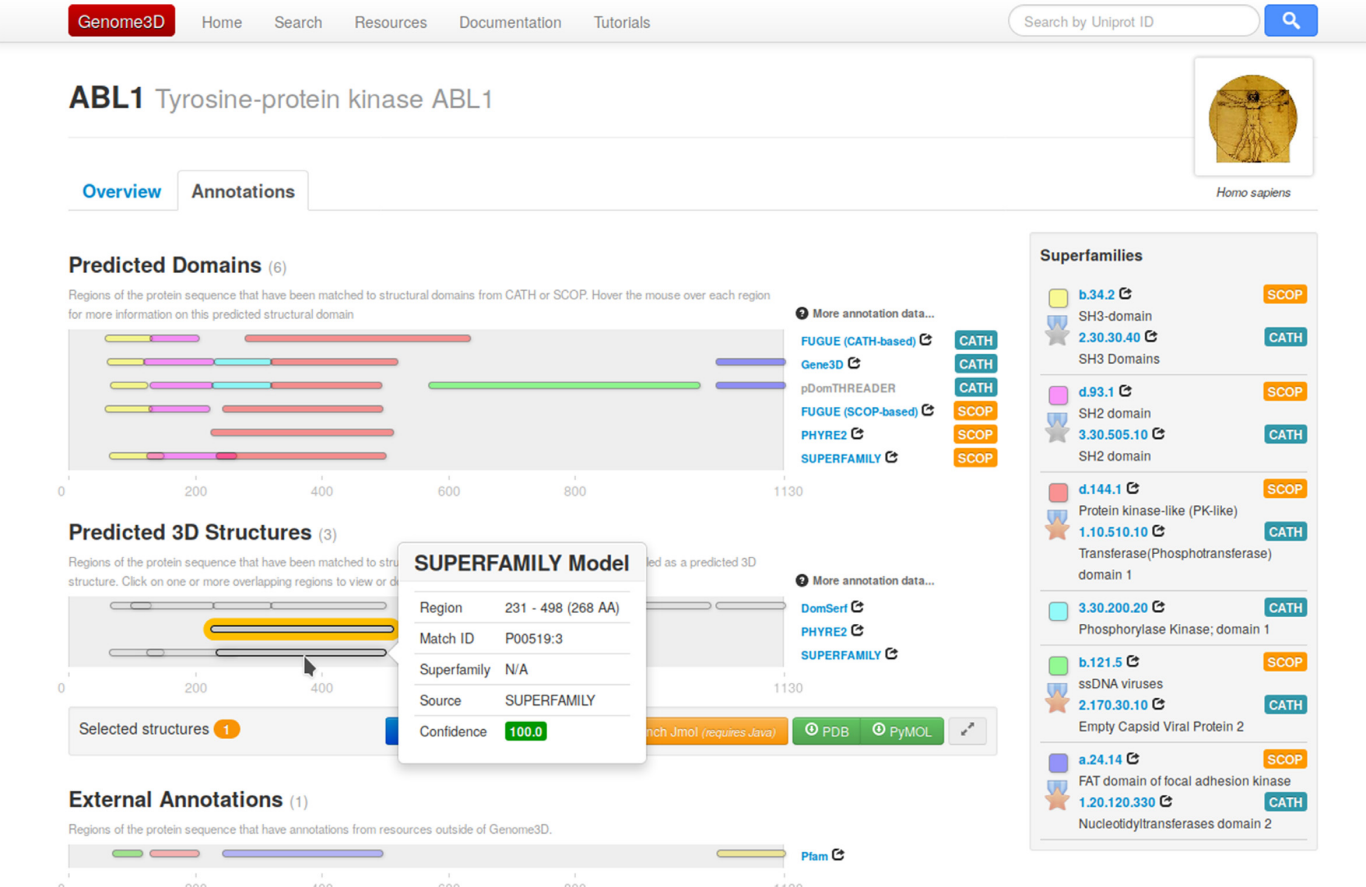
Screenshot showing a typical Genome3D webpage for the gene ABL1_HUMAN. Annotations are grouped into predicted domains (coloured according to the evolutionary relationships within CATH and SCOP) or predicted 3D structures if a group has provided 3D coordinates.

The Genome3D website is typically navigated by searching for a target sequence of interest (by identifier, keyword or sequence similarity). Selecting a particular entry (ie UniProtKB entry) takes the user to a web page that contains an overview of information about the protein with a separate tab displaying all the structural annotations contained in Genome3D. Each annotation is shown as a ‘pill’ signifying a particular region of the protein sequence that has an associated structural prediction (see Figure 1). Predictions are broadly grouped according to whether they correspond to the location of a predicted domain (i.e. high similarity to a structural domain classified in CATH or SCOP) or whether the annotation provides a predicted 3D structure (atomic 3D coordinates). More details on the features of the web pages can be found in previous NAR articles (6,7).

## IMPROVEMENTS

### Improving the mapping between CATH and SCOP

The similarity between a superfamily in CATH and a superfamily in SCOP was quantified by first collecting the superset of all PDB chains containing structural domains in the two superfamilies. Then, for each of these PDB chains, the similarity in domain boundary assignment and the similarity in superfamily assignment was calculated between the two resources. The degree of similarity between two superfamilies deemed equivalent between CATH and SCOP was signified by the categories: Bronze, Silver and Gold (according to increasing similarity of domain boundaries and superfamily membership). Since these classification databases can generate regular releases, it was important that this mapping can be generated automatically. However, since the last NAR update, additional manual curation has identified heuristic rules that can help identify new equivalences, effectively promoting a number of existing relationships from lower to higher categories (eg Bronze to Gold). The current mapping can be summarized as:

- Gold (921 pairs, was 763): SCOP and CATH superfamily display consistently highly similar approaches to both domain boundary assignment and homology detection.
- Silver (179 pairs, was 134): superfamilies are similar but may contain occasional differences in domain boundary assignment and that may have differences between resources in which structures have been classified.
- Bronze (564 pairs, was 532): superfamilies are more similar to each other than to any other superfamily but that may have substantial differences in approaches to domain boundary assignment and/or homology detection.

As a measure of overall coverage, the percentage of all CATH domains that belong to superfamilies within the Bronze, Silver and Gold categories corresponds to 79.5%, 43.6% and 16.5% respectively.

### Refreshing and expanding the shared dataset of protein sequences

The shared set of protein sequences used as annotation targets in Genome3D are selected by querying UniProtKB API with a list of taxon identifiers. The keyword ‘KW-1185’ is added to the search query to ensure that the sequences exist in the reference proteome. For the most recent release, the Genome3D consortium selected four additional model organisms to be annotated: wheat, cow, pig, tuberculosis, bringing the total number of reference proteomes to 14. The percentage of sequences within these organisms that currently have at least one annotation in Genome3D is: 47.2% (wheat), 83.7% (cow), 17.3% (pig), 80.0% (tuberculosis). In addition to these representative proteomes, two additional datasets were chosen to represent protein sequences from unique domain architectures in Pfam (based on single-domain and multi-domain proteins) (7). All resulting datasets were made available on an FTP site for individual resources to download and process (ftp://orengoftp.biochem.ucl.ac.uk/genome3d/).

Information within UniProtKB changes over time (2); entries get modified and in some cases, the primary sequence may change. Many of the target UniProtKB entries in the most recent Genome3D have been annotated in previous releases and annotating groups may decide to reuse existing annotation data if the underlying target sequence has not changed (by matching the md5 digest of the sequence). The released sequence datasets provide additional metadata such as the ‘date of last modification’ and ‘unique md5’ to allow groups to decide for themselves whether to refresh annotations made in previous releases.

**Figure 2.**
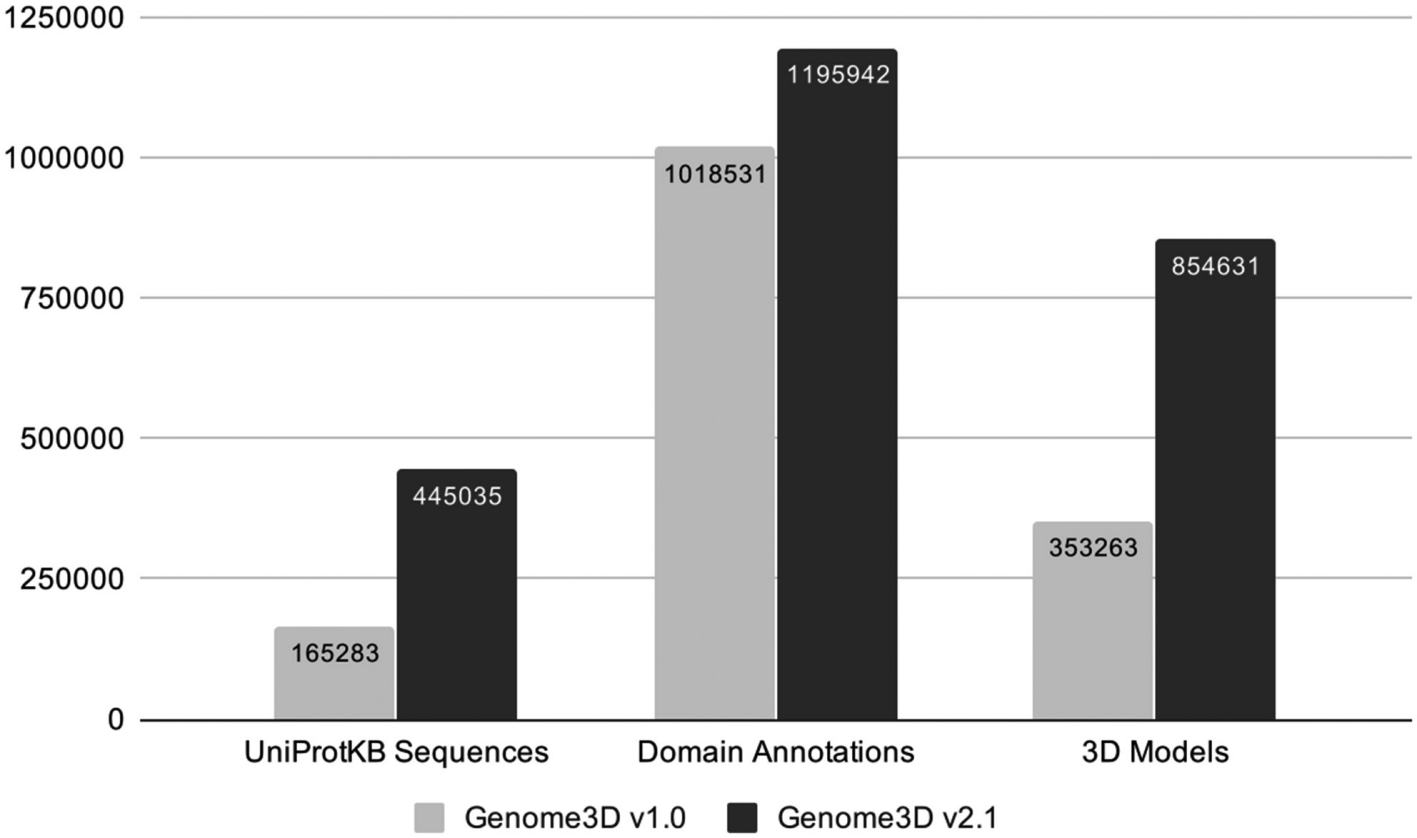
A summary of the increase in target sequences and the number of annotations (split into domain annotations and 3D models) when comparing the latest version of Genome3D v2.1 against the Genome3D release from the previous NAR update article (v1.0, 2015).

The overall increase in target sequences and prediction data for this release of Genome3D is highlighted in Figure 2.

### Using an API to mediate data entry

The process of data submission described in the previous Genome3D NAR article (7) included a number of steps that involved significant manual processing. Annotating groups would initially run their algorithms and make their data available on their own website. Annotations would then be manually downloaded on the server side, then parsed, validated and loaded to the database. Any issues with data validation at this stage would have to be identified, described to the submitting group, fixed, then the data submission process would be repeated.

**Figure 3.**
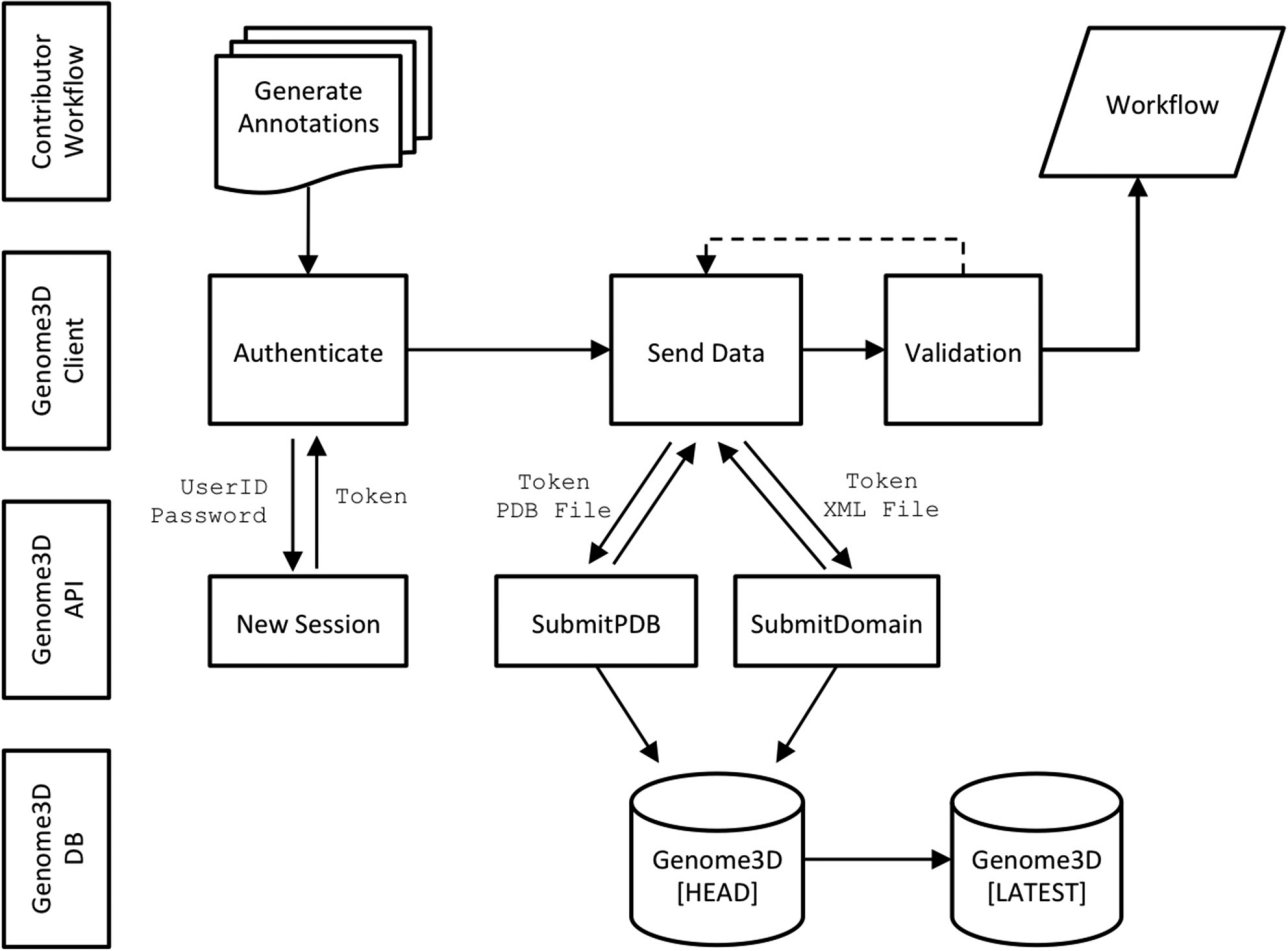
An overview of the workflow for data entry in the new Genome3D API. Key advantages over the previous mechanism include: removing inefficient, time-consuming and error-prone manual processing steps, improving flexibility for resource to submit annotations in their own schedule, allowing annotations to be integrated into existing pipelines and reducing the work involved in adding new annotation groups and algorithms.

In order to make this process more efficient and less dependent on individual human expertise, the manual submission process was replaced by an API that allows groups to ‘push’ their predictions directly to Genome3D (see Figure 3). This mechanism has the advantage that data is validated immediately; the tight feedback loop enabling groups to identify and fix validation issues immediately. Additionally, uploading data via unsupervised API calls provides a mechanism can easily be integrated into existing data pipelines. The API was developed on RESTful principles and is described by an OpenAPI compliant specification, thus providing a platform and language-independent interface (https://www.genome3d.eu/api). For convenience, an example client was made available to allow groups easy access to uploading data to the Genome3D server (https://uclorengogroup.github.io/genome3d-openapi-client/).

Since the API allows remote users to insert, update and delete entries in Genome3D, it was important that groups were only allowed to manage their own data. This authentication is implemented by the ‘password grant’ OAuth2 protocol which authenticates private user credentials and provides an access token that can be added to HTTP headers to authorize all subsequent API requests.

It is useful for the annotating groups to have the flexibility to upload data in their own schedule, however it is also important to provide stable, static releases for subsequent scientific analysis. As a result, three versions of the API and web pages are provided: latest, head and daily (see Table 2).

**Table 2.** Description of Genome3D databases and releases

Release	Permission	Domain	Purpose
LATEST	Read only	https://www.genome3d.eu	Most recent static release
HEAD	Read / Write	https://head.genome3d.eu	Manage annotations for upcoming release
DAILY	Read / Write	https://daily.genome3d.eu	Testing area (refreshed daily from HEAD)

### Exporting Genome3D annotations to InterPro

The InterPro database (http://www.ebi.ac.uk/interpro/) classifies protein sequences into families and predicts the presence of functionally important domains and sites, based on information from 14 member databases, including CATH-Gene3D and SUPERFAMILY. Recently, a new InterPro entry type was introduced named ‘Homologous superfamily’ (described in more detail in (13)). This helps to integrate signatures from CATH-Gene3D And SUPERFAMILY by better reflecting the structural data upon which these models are constructed. The predicted domains and 3D models from Genome3D were not previously represented within InterPro primarily because the process of generating 3D models is too computationally expensive to calculate for the entire UniProt database on a monthly basis (as happens for InterPro member databases). However, it was recognised that Genome3D provides useful supplementary annotations to those already found in InterPro, often adding greater coverage due to the more sensitive threading-based algorithms.

**Figure 4.**
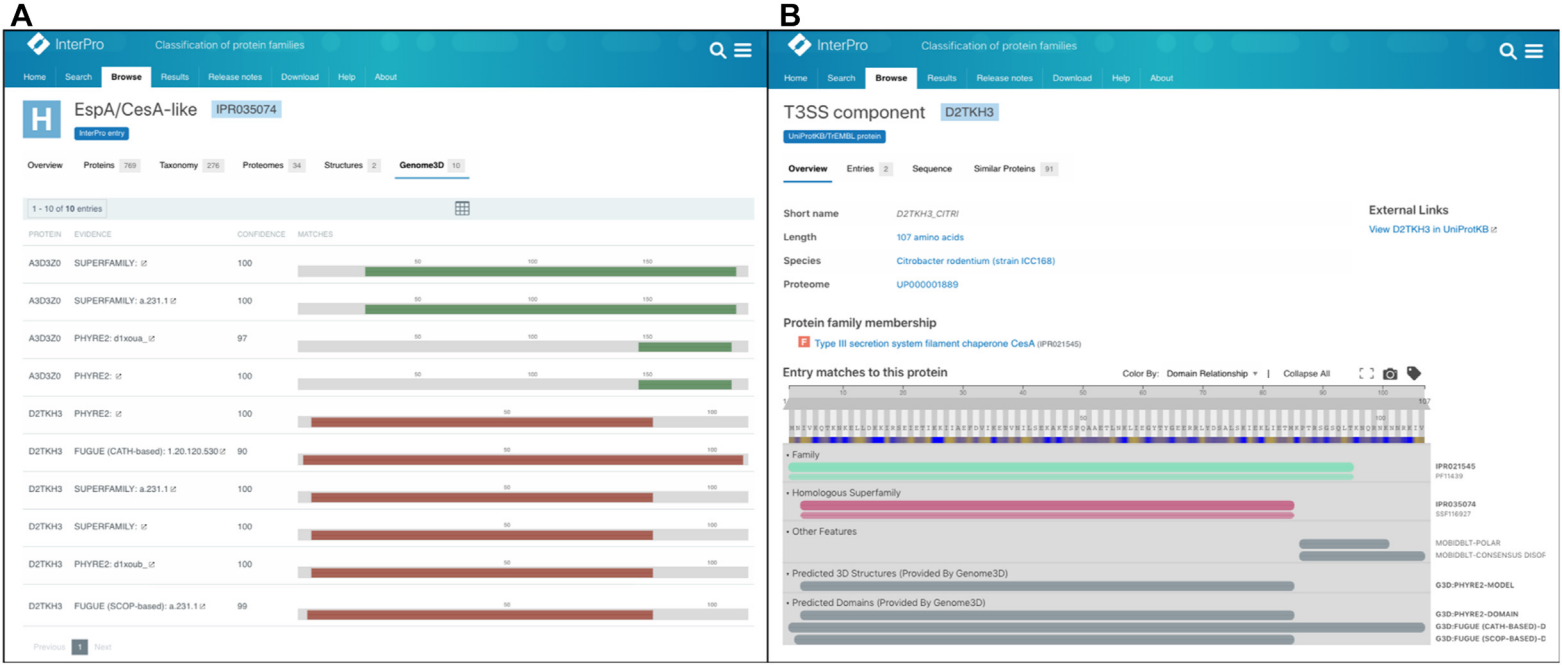
(**A**) Genome3D predictions table for IPR035074. 10 domains have been found for two UniProt accessions. (**B**) D2TKH3 protein page in InterPro. The sequence viewer shows Genome3D domains and 3D structure predictions alongside integrated InterPro entries and unintegrated InterPro member databases.

To address this, the predicted domains and 3D structures as provided by Genome3D have now been made available for a number of specific data types within InterPro: InterPro entry, UniProt sequence and PDB structure. These annotations are dynamically retrieved by the InterPro website using newly created endpoints in the Genome3D API. Genome3D annotations are available within a ‘tab’ of the InterPro entry page. This page provides a list of all the UniProt accessions that contain a Genome3D annotation within the current InterPro entry. Each annotation has information such as the annotating resource/tool, a domain identifier (if available), a confidence score and a visualization of the predicted regions within the protein (Figure 4A). Genome3D annotations are also made available within the ‘Protein’, and ‘Structure’ pages of InterPro, via an extended version of the ProtVista viewer (14). On the Protein page, two features have been added to the existing InterPro tracks found within the Protvisa viewer, namely ‘Predicted 3D Structures’ and ‘Predicted Domains’ (see Figure 4B). Similarly, information is included on the structure page, where sequences found within a structure can be selected and viewed using ProtVista. The integration of the Genome3D annotations at different levels within InterPro supplements the already high coverage of InterPro and increases the visibility of additional structural data contained within Genome3D.

## CONCLUSIONS

Genome3D is a collaborative resource that provides structure-based predictions to help users learn more about their protein sequences. Bringing together annotations from a number of independent prediction algorithms helps to indicate regions of agreement which can be used to assign confidence when considering these predictions in further analysis. The process of normalizing data from different resources has also provided a set of data transfer protocols and formats agreed by a number of groups in the community. The results of this may provide a useful starting point for future projects looking to leverage protein structure annotation data. In addition, Genome3D provides a single point of access for the further dissemination of data to other resources, such as InterPro.

In this context the recently funded 3D-Beacons project, which will be managed by Sameer Velankar at the PDBe (15), will exploit Genome3D to integrate domain based 3D-models from a number of other European and American groups in the future (e.g. SWISS-MODEL, Rosetta-Gremlin). It will also support research into a robust mechanism for weighting each position in a consensus 3D-model (derived by integrating multiple predictions) based on features related to the confidence in individual methods and the agreement between methods.

Implementing data transfer via the Genome3D API has greatly improved the overall efficiency for both the client (i.e. annotating groups) and server (i.e. Genome3D). Making it possible to add this process into existing automated pipelines reduces the maintenance requirements (in terms of time and technical skills) for annotating groups, thus making the continuation of the Genome3D resource more feasible in the long term. It has also simplified the process of adding new prediction groups and algorithms to the resource. Indeed, one of the desired outcomes of this article is to encourage additional groups from the structure prediction community to get in touch and add their own annotations.

## DATA AVAILABILITY

All Genome3D data is freely available. Datasets of target protein sequences are available to download via FTP (ftp://orengoftp.biochem.ucl.ac.uk/genome3d). Annotations can be downloaded via the API (https://www.genome3d.eu/api). A tool to help users interact with the Genome3D API from the command line is available on GitHub (https://github.com/UCLOrengoGroup/genome3d-openapi-client).
